# An infant with refractory cytomegalovirus‐induced thrombocytopenia

**DOI:** 10.1002/ccr3.2581

**Published:** 2019-12-17

**Authors:** Suguru Uemura, Takeshi Mori, Nanako Nino, Nana Sakakibara, Satoru Takafuji, Shota Myojin, Yuichi Takami, Ichiro Morioka, Noriyuki Nishimura, Masaaki Kugo, Kazumoto Iijima

**Affiliations:** ^1^ Department of Pediatrics Kobe University Graduate School of Medicine Kobe Japan; ^2^ Department of Pediatrics Japanese Red Cross Society Himeji Hospital Himeji Japan; ^3^ Department of Pediatrics and Child Health Nihon University School of Medicine Itabashi‐ku Japan

**Keywords:** cytomegalovirus, infant, intravenous immunoglobulin, thrombocytopenia

## Abstract

The present case underscores the importance of considering the association of severe thrombocytopenia or immune thrombocytopenia with cytomegalovirus (CMV) infection because CMV‐induced thrombocytopenia occasionally requires antiviral therapy.

## Graphical abstract

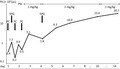

## 1 BACKGROUND

Most cases of cytomegalovirus (CMV) infection are asymptomatic or self‐limited in immunocompetent patients. However, CMV infection occasionally induces severe thrombocytopenia.^1^, ^2^, ^3^, ^4^, ^5^, ^6^ Immune thrombocytopenia (ITP) is an autoimmune disease characterized by isolated thrombocytopenia due to the inhibition of platelet production and destruction of existing platelets.^1^ Moreover, it is the most common cause of pediatric thrombocytopenia.^1^ Although the etiology of ITP remains unknown in most cases, it may be triggered by viral infections such as by CMV or by other immunological and environmental agents.^7^ Severe and life‐threatening hemorrhage is very rare (<1.0%) in such cases.^8^, ^9^ CMV‐related secondary ITP often occurs a few weeks after the onset of CMV infection symptoms such as fever, elevated liver enzymes, hepatosplenomegaly, and cervical lymphadenopathy, because of the occurrence of underlying immunological reaction during this period.^3^, ^4^ Meanwhile, CMV infects megakaryocytes directly, causing CMV infection symptoms at the time of thrombocytopenia diagnosis. This mechanism is regarded as CMV‐induced thrombocytopenia.^3^, ^4^

It is difficult to distinguish CMV‐induced thrombocytopenia from CMV‐related secondary ITP. However, early detection of CMV infection is paramount because CMV‐induced thrombocytopenia occasionally requires antiviral therapy.^3^ Herein, we report a case of refractory CMV‐induced thrombocytopenia in a 38‐day‐old infant.

## 2 CLINICAL CASE REPORT

A 38‐day‐old boy was admitted to our hospital because of the presence of petechiae from trunk to legs and oral wet purpura. His mother was a 35‐year‐old woman (gravid 2, para 2) with no remarkable medical history. Her pregnancy course was uncomplicated, and her platelet count was also normal during pregnancy. There were no hemorrhagic episodes during the perinatal period of the boy, and he was born at full term via aspiration delivery. His weight at birth was 3304 g. The baby was breast‐fed and had no remarkable family history. He underwent a medical check‐up on the 31st day after birth, which revealed no particular problems. At admission, he was afebrile with blood pressure of 98/60 mm Hg, pulse of 142/min, respiratory rate of 36/min, and O_2_ saturation of 100% on room air. He had neither hepatomegaly nor splenomegaly. A laboratory examination showed a white blood cell count of 23 600/µL with 11.0% atypical lymphocytes, CD4‐positive cell count of 1044/µL, a hemoglobin level of 11.9 g/dL, and platelet count of 4000/µL. Immature platelet fraction was normal (2.9%). CD4/CD8 ratio was also normal (0.82). The size of platelet was normal. The aspartate aminotransferase and alanine aminotransferase levels were 59 IU/L and 55 IU/L, respectively. Immunoglobulin G (IgG), IgA, and IgM were 773, 21, and 50 mg/dL, respectively. A coagulation test was normal. His chest/abdominal CT findings were normal.

**Figure 1:**
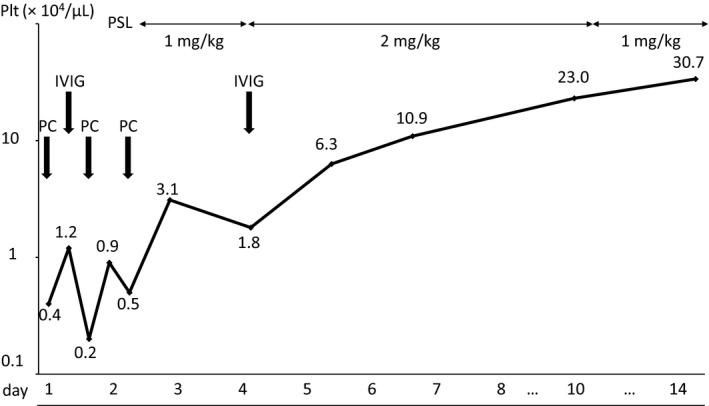
Clinical course of our patient. IVIG, intravenous immunoglobulin; PC, platelet transfusion; Plt, platelet count; PSL, prednisolone

Platelet transfusion was performed after admission because ITP was not suspected due to his age. However, his platelet count was only 12 000/µL after platelet transfusion (Figure 1). IVIG (1 g/kg) was subsequently administered because of the refractoriness to transfusion, which was suspected to be due to ITP. Despite IVIG administration, his platelet count decreased to 2000/µL. Although another platelet transfusion was performed, his platelet count increased only to 9000/µL. A subsequent bone marrow examination showed a nuclear cell count of 105 000/µL without morphologically abnormal cells and megakaryocyte count of 8/µL. After the exclusion of malignant diseases, prednisolone (PSL; 1 mg/kg) was administered as a second‐line therapy for ITP with another platelet transfusion. After the initiation of PSL therapy, his platelet count increased to 31 000/µL, but decreased to 18 000/µL after 2 days. CMV infection was diagnosed based on the presence of CMV IgM (1.64) and IgG (14.2), and the results of a CMV antigenemia assay using monoclonal antibodies C10/C11 (4 positive cells/ 150 000 leukocytes) at admission. IVIG (1 g/kg) were re‐administered, and PSL dose was increased to 2 mg/kg. Thereafter, his platelet count gradually normalized. Thrombocytopenia has not recurred after the discontinuation of PSL. The patient has been normal development, without recurrence, for more than 6 months after onset. Using quantitative PCR analysis, CMV‐DNA was detected in his urine after treatment but not in his dried umbilical cord. Based on these results, thrombocytopenia was associated with acquired CMV infection. No abnormalities were observed on brain magnetic resonance imaging. Auditory brainstem responses revealed normal waves from I to V at 90 dB and V wave was detected at 30dB. These clinical findings indicate that the patient was infected with CMV after birth, excluding a congenitally acquired infection.

## 3 DISCUSSION

Herein, we described a 38‐day‐old boy with CMV‐induced thrombocytopenia. Our initial diagnosis was ITP. Retrospectively, however, CMV‐induced thrombocytopenia is more suitable in the patient's diagnosis, because immune system and lymphoid activation are immature for his age and the general peak occurrence of ITP in childhood is between 2 and 5 years of age.^10^

In a 38‐day‐old infant, either congenital or acquired CMV infection can occur. Thrombocytopenia often occurs in patients with congenital CMV infection.^11^ In our case, quantitative PCR did not reveal CMV‐DNA in his dried umbilical cord at birth,^12^ indicating acquired CMV infection. The detection of CMV‐DNA in the dried umbilical cord is useful to prove congenital CMV infection.^13^ In preterm babies, acquired CMV infection can occur through breast milk because of the low level of anti‐CMV antibodies transmitted via the placenta.^14^ Because the status of maternal CMV infection was not evaluated, the infection route of CMV remained to be determined in our case. CDC recommends that congenital CMV infection should be diagnosed based on the presence of CMV‐DNA in the urine, saliva, or blood, within 3 weeks after birth. Infection cannot be diagnosed using tests that detect antibodies to CMV.^15^ Moreover, congenital CMV infection cannot be diagnosed using samples collected more than three weeks after birth because testing after this time cannot distinguish between a congenital infection and an infection acquired during or after delivery.^15^ In our case, we could not access other samples within 3 weeks after his birth to detect congenital CMV infection and the possibility of false‐negative results of CMV in the dried umbilical cord cannot thus be ruled out. Therefore, we could not rule out congenital CMV infection completely.

Thrombocytopenia in neonates is relatively common, occurring in 1%‐5% of healthy term infants and in 20%‐50% of sick infants. Frequent etiologies include sepsis, disseminated intravascular coagulation, respiratory distress syndrome, and maternal factors, among others.^16^ Thrombocytopenia due to maternal factors is caused by ITP, systemic lupus erythematosus, and drugs.^16^ However, in our case, his mother showed no problems during pregnancy, did not receive any thrombocytopenia‐inducing drugs, and did not show any remarkable medical history. Her platelet count was also normal. Therefore, we judged that there was no association between thrombocytopenia and maternal causes in this case.

Neonatal alloimmune thrombocytopenia (NAIT) is the most common cause of severe thrombocytopenia in newborns.^17^ Typical patients with NAIT develop petechiae within minutes of birth, followed by ecchymosis and cephalohematoma.^17^ After delivery, the greatest risk of hemorrhage is in the first 96 hours of life. Thrombocytopenia in patients with NAIT resolves naturally within 1‐2 weeks after birth.^17^ Because there were also no hemorrhagic episodes in his perinatal period, we judged that there was no association between thrombocytopenia and NAIT in this case, although anti‐human platelet antigen alloantibodies were not tested.

In the retrospective analysis, there were no significant differences between CMV‐induced thrombocytopenia and CMV‐related secondary ITP in terms of age, sex, and platelet count at diagnosis.^3^ Interestingly, antiviral therapy was required in 8 of the 23 patients with CMV‐induced thrombocytopenia, while no patients with CMV‐related secondary ITP required antiviral therapy. Furthermore, some patients with CMV‐induced thrombocytopenia showed complicated clinical courses such as refractory or chronic courses, compared with CMV‐related secondary ITP. The data of bone marrow examination did not give new knowledge about CMV‐induced thrombocytopenia and CMV‐related secondary ITP, because a few patients underwent bone marrow examination.

In conclusion, CMV should be evaluated in suspected cases of refractory thrombocytopenia because CMV infection occasionally induces severe and refractory thrombocytopenia, requiring antiviral therapy.

## CONFLICTS OF INTEREST

The authors have no conflicts of interests to declare.

## AUTHOR CONTRIBUTIONS

SU, TM, NN, NS, ST, SM, YT, and MK: participated in patient's evaluation and treatment. SU, TM, IM, NN, MK, and KI: reviewed and revised the manuscript. All authors read and approved the final manuscript.
